# Atomic-scale view of the photoinduced structural transition to form *sp*^3^-like bonded order phase in graphite

**DOI:** 10.1038/s41598-023-47389-x

**Published:** 2023-12-15

**Authors:** Eiichi Inami, Keita Nishioka, Jun’ichi Kanasaki

**Affiliations:** 1 School of Systems Engineering, Kochi University of Technology, 185 Miyanokuchi, Tosayamada, Kami, Kochi 782-8502 Japan; 2 Math. and Science Education Research Center, Kanazawa Institute of Technology, 7-1 Ohgigaoka, Nonoichi, Ishikawa 921-8501 Japan; 3 Graduate School of Engineering, Osaka Metropolitan University, 3-3-138 Sugimoto, Sumiyoshi-ku, Osaka, 558-8585 Japan

**Keywords:** Engineering, Materials science, Mathematics and computing, Nanoscience and technology, Physics

## Abstract

Photoexcitation of solids often induces structural phase transitions between different ordered phases, some of which are unprecedented and thermodynamically inaccessible. The phenomenon, known as photoinduced structural phase transition (PSPT), is of significant interest to the technological progress of advanced materials processing and the fundamental understanding of material physics. Here, we applied scanning tunnelling microscopy (STM) to directly characterise the primary processes of the PSPT in graphite to form a *sp*^3^*-*like carbon nano-phase called *diaphite*. The primary challenge was to provide microscopic views of the graphite-to-*diaphite* transition. On an atomic scale, STM imaging of the photoexcited surface revealed the nucleation and proliferation processes of the *diaphite* phase; these were governed by the formation of *sp*^3^-like interlayer bonds. The growth mode of the *diaphite* phase depends strongly on the photon energy of excitation laser light. Different dynamical pathways were proposed to explain the formation of a *sp*^3^-like interlayer bonding. Potential mechanisms for photon-energy-dependent growth were examined based on the experimental and calculated results. The present results provide insight towards realising optical control of *sp*^2^-to-*sp*^3^ conversions and the organisation of nanoscale structures in graphene-related materials.

## Introduction

Exploring the hidden multistability of structural phases and the associated dynamical pathways connecting different ordered phases is essential for understanding condensed matter and technological breakthroughs in material development. It is known that the nonequilibrium electron–phonon dynamics of electronically excited states can trigger structural instability in the matter, which often results in phase transitions between phases of different orders^1–3^. Optical excitation techniques have opened novel dynamic pathways for phase transitions between several orders of phases with differing structural and electronic properties^4–11^. The present authors previously discovered that visible fs laser excitation causes the photoinduced structural phase transition (PSPT) from the stacking *sp*^2^-bonded phase of graphite to the *sp*^3^-like bonded metastable phase, named ‘*diaphite*’^11^. The *diaphite* phase is characterised by a twisted structure, including periodically arranged *sp*^3^-type interlayer bonds, which conventional thermodynamic processes cannot achieve. According to several reports, the photoexcitation of carbon materials triggers *sp*^2^–*sp*^3^ conversion, resulting in different forms of PSPTs and related changes in the physical properties^12–17^. However, the mechanism of action of PSPTs has not been completely elucidated.

PSPT generally proceeds through two stages: the atomic-scale nucleus is first triggered, and the new order phase proliferates around it^18–22^. Thus far, ultrafast techniques, such as time-resolved electron/X-ray crystallography^4–9,16,23–30^ and time-resolved optical spectroscopy^10,29–33^, have been used to understand the fundamental processes involved in PSPTs. However, a microscopic view of these processes is inaccessible because these studies provide ensemble-averaged structural/electronic order information. Therefore, the key issues to be resolved are to elucidate the atomic processes underlying nucleation, followed by the proliferation of the photoinduced ordered phase, and to determine the key factors governing these processes. This study used scanning tunnelling microscopy (STM) to obtain real-space images and spectroscopic information with atomic resolution concerning the evolution of the graphite-to-*diaphite* (G-to-D) transition. Atomic-scale imaging of a photoexcited graphite surface revealed a series of intermediate nanostructures produced during the G-to-D transition: the formation of a nucleus involving an interlayer bond of weak *sp*^3^-nature and the subsequent proliferation of interlayer-bonded domains from the nucleus, eventually forming the *diaphite* phase. Furthermore, we revealed that the formation of nuclei and proliferation of the *sp*^3^-bonding domain show strong photon energy-dependent efficiencies, demonstrating the different dynamical pathways associated with the two processes. Possible mechanisms of photon energy-dependent growth are proposed based on the experimental and calculated results. These findings establish a fundamental concept underlying the precise control of PSPT at the atomic scale, advancing optical material processing techniques.

**Figure 1:**
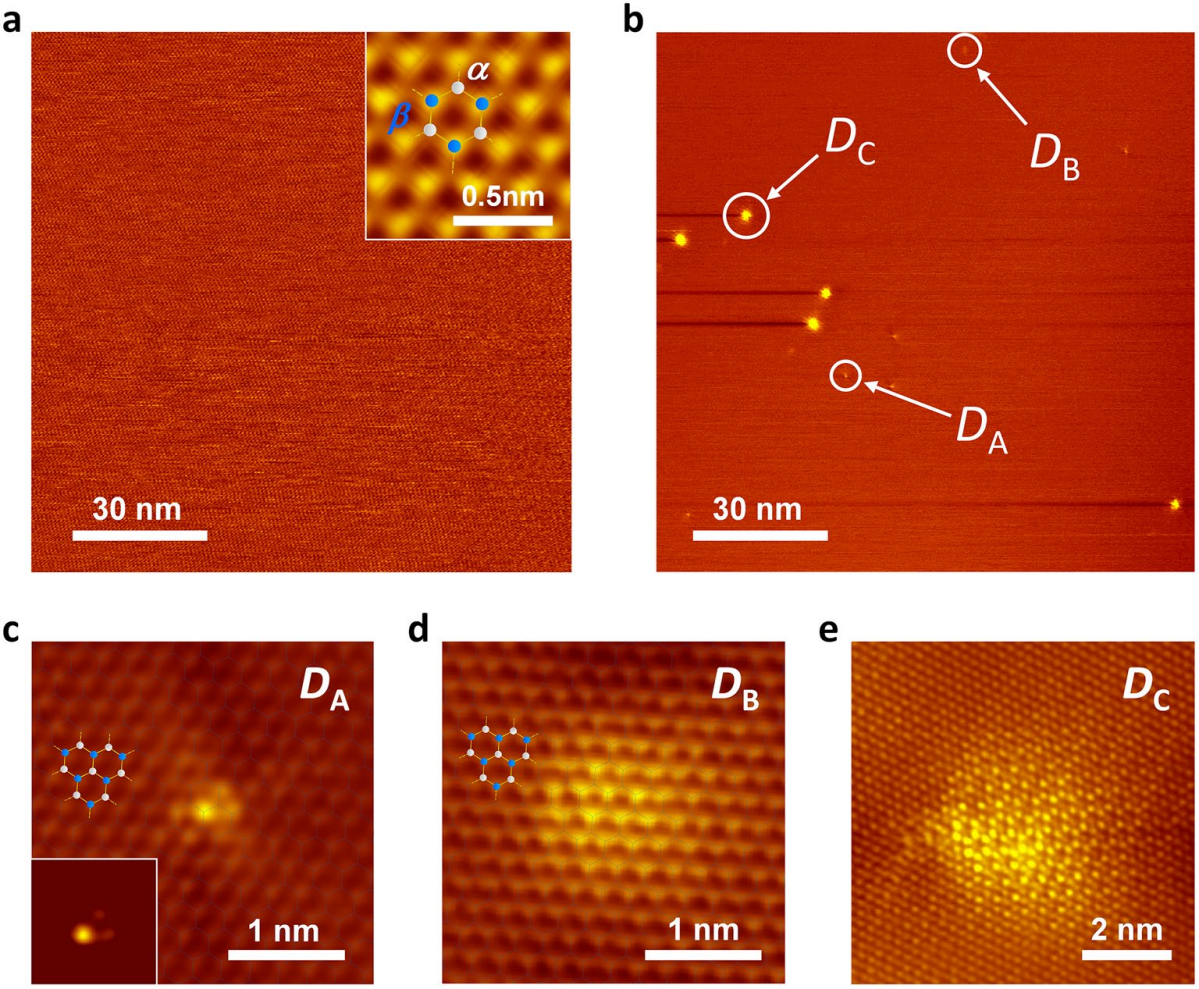
Femto-second laser-induced nanostructure in graphite. (**a**,**b**) Quasi-constant-height STM image showing wide-scale views (120 nm × 120 nm) of the surface before (**a**), and after 1 × 10^5^ shots of *p*-polarized 800 nm laser pulses at 77 mJ/cm^2^ (**b**). Sample biases were set at *V*_S_ = 0.3 V. The inset in (**a**) shows an atomic-resolution image superimposed by a honeycomb lattice structure of graphite with basis atoms *α* (white) and *β* (blue). Dark lines tailing from the bright spots in (**b**) are artifacts caused by the feedback error due to the quasi-constant height mode scans. (**c**–**e**) Magnified images of the photoinduced domains labelled *D*_A_, *D*_B,_ and *D*_C_ in (**b**), respectively. The honeycomb lattices in (**c**) and (**d**) represent the carbon atom sites on pristine graphite. Sample biases were set at *V*_S_ = − 0.3 V for (**c**), *V*_S_ = − 0.5 V for (**d**), and *V*_S_ = − 0.1 V for (**e**). The inset of (**c**) shows the same image as (**c**), but the original contrast of the pristine graphite was subtracted.

Clean surfaces of highly oriented pyrolytic graphite (HOPG) in an ultra-high vacuum (UHV) chamber (base pressure < 7 × 10^−9^ Pa) were illuminated with 120 fs laser pulses of 800, 400, and 266 nm, and the induced structural changes were directly observed using STM (see details in the “Methods” section). Figure 1a shows a large-scale STM image (120 nm × 120 nm) of the HOPG surface before laser excitation. A single domain characterised the surface without any irregularities over a wide terrace, where a honeycomb structure composed of two different atomic sites, α- and β-carbon sites^34^, is well-defined (inset of Fig. 1a). Excitation with 1 × 10^5^ laser pulses (800 nm) at a fluence of 77 mJ/cm^2^ results in the formation of several nanoscale structures on the surface (Fig. 1b). Enlarged views of the typical nanostructures, labelled *D*_A_, *D*_B_, and *D*_C_, are shown in Fig. 1c–e. Structure *D*_A_ (Fig. 1c) is the smallest structure observed, consisting of one bright spot and surrounding less bright spots (inset of Fig. 1c). In contrast, in structure *D*_B_ (Fig. 1d), the domain of 1–5 nm in size exhibited brighter spots compared to the intact graphite sites (Other examples of *D*_B_-type structures can be found in Fig. S1 of the supplementary information). As shown in the STM images of *D*_A_ and *D*_B_, the spots were arranged completely on the original lattice sites of the pristine graphite, revealing negligible in-plane displacement of the surface atoms. Conversely, in structure *D*_C_ (Fig. 1e), the domain of size greater than 5 nm shows a periodic buckling structure accompanied by shear-type displacements of STM spots from the original lattice sites. These structural features align with those of the *diaphite* phase reported previously^11^. Photoinduced nanostructures, divided into three types, were also found upon excitation at other wavelengths (400 and 266 nm). Thus, the present results demonstrate that the photoinduced G-to-D transition occurs over a broad photon energy range.

**Figure 2:**
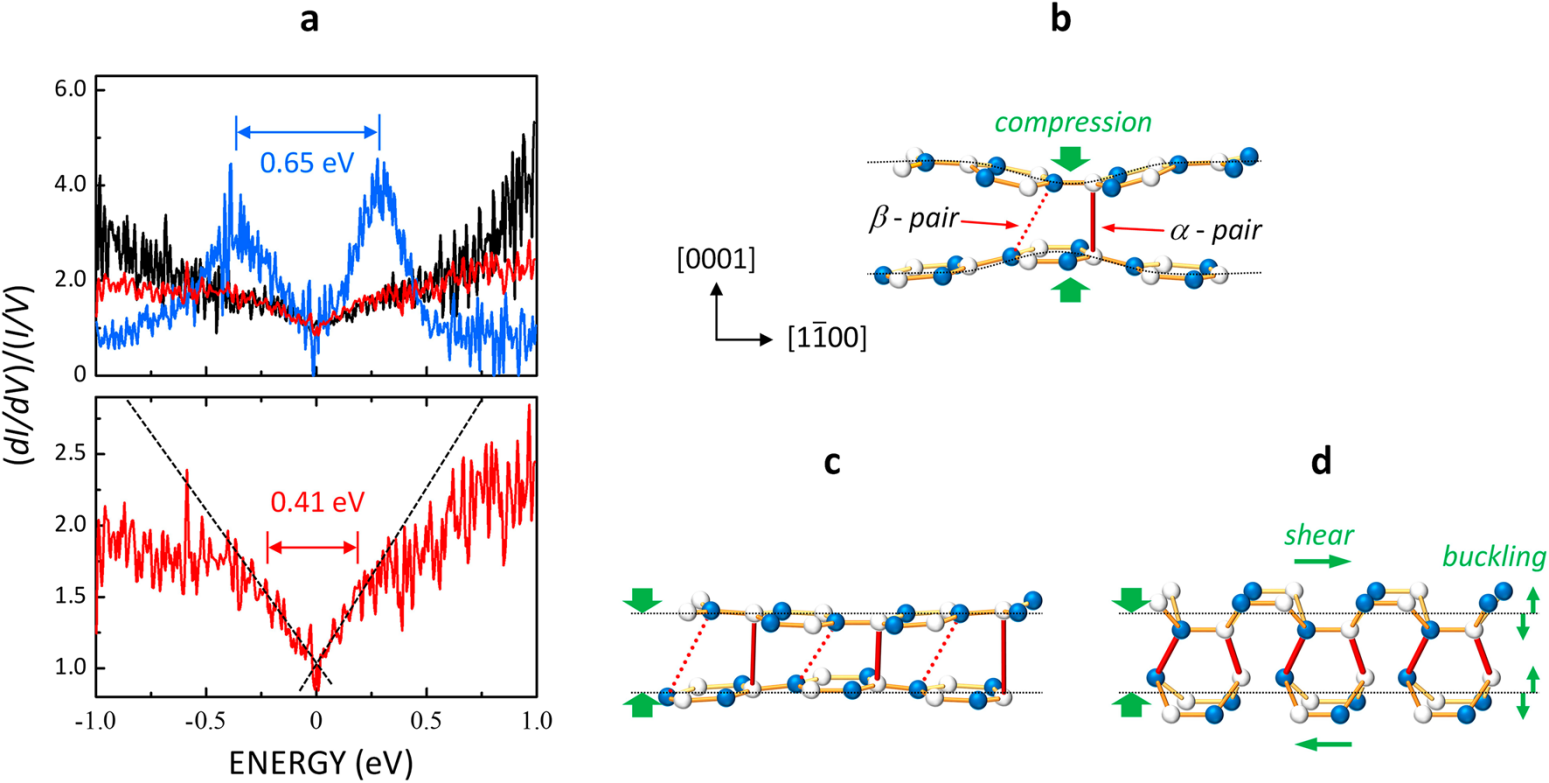
Electronic structures and atomic arrangement of interlayer bonded domains. (**a**) (Upper panel) (*dI*/*dV*)/(*I*/*V*) spectra acquired for the pristine graphite surface (black), *D*_A_ (red), and *D*_C_ (blue)^11^. (Lower panel) (*dI*/*dV*)/(*I*/*V*) spectra acquired on the *D*_A_ at an expanded scale. The dashed lines in (**a**) represent the linear fit of dispersion. The Fermi level is referenced to zero energy. (**b**–**d**) Structural models of laser-induced nanostructures, *D*_A_ (**b**), *D*_B_ (**c**), *D*_C_ (**d**). White and blue balls represent α- and β-carbon atoms, respectively.

The local density of states (LDOS) spectrum, (*dI/dV*)/(*I/V*), was measured for *D*_A_ and compared with those for *D*_C_ and pristine graphite in Fig. 2a^11^. The LDOS of graphite (black) exhibited a V-shape with no discernible features consistent with its semi-metallic electronic structure. Conversely, the *D*_C_-type structure (blue) showed well-defined peaks at − 0.35 and 0.3 eV. As reported in Ref.^11^, the LDOS of *D*_C_ agrees with the calculated value expected for the *diaphite* structure shown in Fig. 2d. The observed peaks were attributed to the band gap formed by the excitation-induced *sp*^3^-like interlayer bonds. In contrast, the LDOS of the *D*_A_-type structure (red) differs from those of the other two structures. As evident from the magnified view (lower panel in Fig. 2a), the LDOS curve resembled that of graphite (dashed line). As the energy moves away from the Fermi level (zero), two breaks appear at − 0.24 and 0.17 eV, from which the two curves deviate. The energy separation between the two breaks (0.41 eV) was 37% smaller than the peak separation of *D*_C_ (0.65 eV). Compared to the LDOS in the *diaphite* phase, the breaks observed for *D*_A_ could be attributed to the formation of interlayer bonds of a reduced *sp*^3^ bonding nature shown in Fig. 2b (this will be discussed in more detail later).

**Figure 3:**
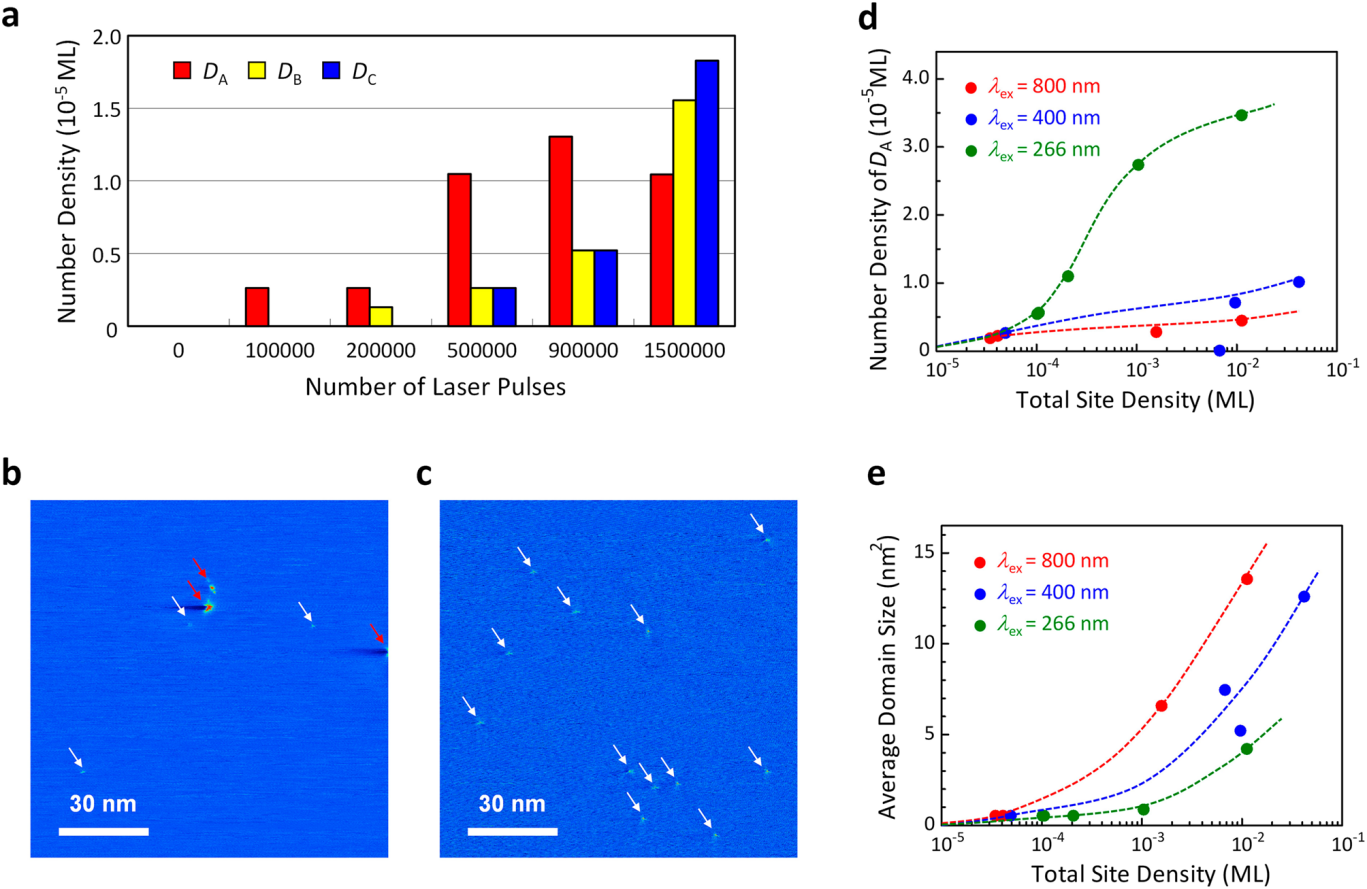
Growth of laser-induced carbon nanostructure in graphite. (**a**) Number densities of *D*_A_, *D*_B_, and *D*_C_ as functions of the number of *p*-polarized 800 nm laser pulses at 60 mJ/cm^2^. (**b**,**c**) Quasi-constant-height STM images showing wide-scale views (120 nm × 120 nm) of the surfaces after 1 × 10^5^ shots of *p*-polarized 800 nm laser pulses at 67 mJ/cm^2^ (**b**), and 266 nm laser pulses at 71 mJ/cm^2^ (**c**). In these images, blue and red regions represent lower and higher current intensities, respectively. The red arrows in both (**b**) and (**c**) indicate *D*_C_-type nanostructures, while white arrows indicate *D*_A_- or *D*_B_-type nanostructures. (**d**) Number densities of *D*_A_ as a function of total site density for 800 nm (red), 400 nm (blue), and 266 nm (green) laser excitation. (**e**) Average domain sizes of the photoinduced nanostructures as a function of the total site density for 800 nm (red) 400 nm (blue), and 266 nm (green) laser excitations. Dashed lines in (**d**) and (**e**) represent visual guides.

To quantitatively investigate the formation and growth processes of the three nanostructures, we conducted STM observations over an area of 360 × 360 nm excited with 800 nm light at several different excitation doses. Figure 3a shows the number densities of *D*_A_-, *D*_B_-, and *D*_C_-type structures as a function of the excitation dose. The number density is defined as the number density of the nanostructures, irrespective of their size, relative to the total number of lattice sites in the probed region. In the early stage of irradiation, the *D*_A_ is the primary product. In contrast, the early stage is an incubation one for *D*_B_ since the growth of *D*_B_ shows a clear delay with respect to that of *D*_A_. Subsequently, *D*_C_ is generated after some delay in a higher irradiation stage where the presence of *D*_A_ decreases. Similar results were obtained for excitations at 400 and 266 nm. Hence, the primary step in the G-to-D transition involves the formation of a *D*_A_-type nanostructure as a nucleus. The delayed growth of *D*_B_-type structures was attributed to the subsequent proliferation of the photoinduced domain from the nucleus, resulting in a decreased number density of *D*_A_-type nanostructures. Under repeated irradiation, the domain further proliferates and eventually transforms into the *diaphite* phase (*D*_C_), accompanied by periodic buckling and shear-type in-plane displacement of the atomic layers (Fig. 2d)^11^.

In the second stage, following the nucleation process, interlayer-bonded domains proliferate from the nuclei, resulting in a change in the size of the photoinduced nanostructures. In Fig. 3b,c, we compared typical large-scale STM images (120 nm × 120 nm) of the surface irradiated by laser pulses at two different wavelengths. The morphologies of nanostructures reveal notable difference between two excitation wavelengths: for 800 nm excitation (Fig. 3b), three out of seven recognized nanostructures are the *D*_C_-type, whereas for 266 nm (Fig. 3c), all twelve products are *D*_A_-type structure. To obtain further insight into the wavelength-dependent morphology, we performed atomically resolved STM imaging after excitations at three different wavelengths. This enables us to measure the number densities of nuclei (*D*_A_) and the average domain size of the photoinduced nanostructures, providing insight into the evolution process at the three excitation wavelengths. The results are plotted in Fig. 3d,e as a function of the total site density (the total number of atomic sites involved in the photoinduced nanostructures relative to the total number of lattice sites in the probed area). As evident in Fig. 3d, the number density of *D*_A_ was much higher under 266 nm excitation than under 400 and 800 nm excitations. In contrast, Fig. 3e shows that the average domain size becomes largest for 800 nm excitation, indicating that the proliferation of interlayer-bonded domains is strongly enhanced by 800 nm excitation. The average domain size decreased as the photon energy of the excitation light increased. Thus, the growth mode in the G-to-D transition was quite different depending on the photon energy: the nucleus was effectively formed at 266 nm excitation, whereas the growth of the photoinduced domain was highly promoted at 800 nm excitation. The present results strongly demonstrate that different dynamic pathways are associated with the nucleation and proliferation processes in the G-to-D transition.

As shown in Fig. 1c–e, the local bonding geometry changed during the sequential formation of the nucleus (*D*_A_), intermediate nanostructure (*D*_B_), and *diaphite* phase (*D*_C_). The STM images of *D*_A_ and *D*_B_ structures (Fig. 1c,d) showed neither modulated periodicity nor shear-type distortion. Thus, the in-plane atomic displacement was negligible when the domain size was limited. In contrast, the *diaphite* phase (Fig. 1e) is characterised by shear-type in-plane displacements relative to the original lattice in HOPG and the buckling of in-plane atoms^11^. By combining STM and density functional calculations, we previously determined the structure of the *diaphite* domain based on the balance between the energy gain owing to the formation of *sp*^3^-like interlayer bonds and the energy loss attributed to local lattice distortions such as (i) interlayer compression, (ii) periodic buckling in each layer, and (iii) in-plane shear displacement of each adjacent layer (Fig. 2d). The formation of *sp*^3^-like interlayer bonds induces lattice distortions, which occur within the *diaphite* domain and along the domain boundary. The *diaphite* domains include many interlayer bonds, which can compensate for the energy loss attributed to the wide-range lattice distortion.

In contrast to the cases of *D*_C_, the shear displacements in *D*_A_- and *D*_B_-type structures are too small to be recognised. One may infer that these precursors include no *sp*^3^-like interlayer bonds, but the atoms are arranged in a two-dimensional honeycomb lattice formed by in-plane *sp*^2^-bonds. Nevertheless, the breaks in the LDOS of the *D*_A_ structure (Fig. 2a) indicate that these nanostructures possess interlayer bonds with *sp*^3^ nature (Fig. 2b,c). However, the smaller buckling corrugation and narrow bonding-antibonding energy split strongly suggest that the interlayer bonding is much weaker in the *D*_A_ structure than in the *D*_C_ structure. Ohnishi et al.^20^ used the ab initio local density approximation theory to determine a metastable configuration of the minimal *diaphite* structure based on the balance between the electronic energy gain due to the formation of a few interlayer bonds and the energy loss due to the inward distortion of the carbon atoms associated with the bonding. As shown in Fig. 2b, the predicted structure includes two different interlayer bonds with a local shear displacement of 0.002 Å: a single interlayer bond between the carbon atoms of an *α*-pair and a weak interlayer bond between the atoms of a *β*-pair adjacent to the *α*-pair bonding. This configuration is stable against the thermal fluctuation at room temperatures and agrees with the observed characteristics of *D*_A_ structures showing a small-sized and anisotropic geometry around the brightest spot on an α site (inset in Fig. 1c) with undetectable lateral displacement. Thus, it is probable that the *D*_A_ structure corresponds to a minimal *diaphite* structure consisting of two interlayer bonds with a weakly *sp*^3^-bonding nature. Similarly, the brighter spots in the *D*_B_-type structure were arranged in their original positions, indicating that the local geometry of weak *sp*^3^-type interlayer bonds is almost the same as that of *D*_A_-type ones, as shown in Fig. 2c.

As described in the previous sections, the photoinduced G-to-D phase transition proceeds through two processes: (1) the formation of nuclei, including weak *sp*^3^-hybridised interlayer bonds at intrinsic lattice sites, and (2) the subsequent aggregation of interlayer bonds around the nuclei to convert them into *diaphite* domains. Our finding of particular interest is that the size distribution of the induced interlayer bonded domains strongly depends on the photon energy of the excitation light. These results demonstrate that the two processes occur through different dynamic pathways. According to ref. 11, interlayer bond formation is induced explicitly by *p*-polarised light, which has an electric vector component parallel to the *c*-axis. Consequently, the formation of interlayer bonds was attributed to charge transfer excitation between the two neighbouring graphene layers. The electron–hole (e–h) pairs generated independently in the adjacent layers can self-localise at the nearest atomic sites. This localisation imparts kinetic energy to the two carbon atoms, causing them to approach each other closely. Based on the molecular dynamics (MD) calculations, Nishioka et al.^21^ have predicted that an excitation energy, ${E}_{\mathrm{e}}$, of 4.5 eV is needed to form a nucleus including a *sp*^3^-like interlayer bond at the e–h localised site. An alternate way to form a nucleus at the original lattice site is through a cooperative excitation effect, in which several self-localised states cooperatively form an interlayer bond at a nearby lattice site^22^. Calculations for some model cases have demonstrated that the effect can form a nucleus even if ${E}_{\mathrm{e}}$ imparted to each self-localised state was as low as 2.5 eV. Thus, two dynamic pathways can form a nucleus at the original lattice site (Process 1). Both pathways were available in the case of 266 nm (4.66 eV) excitation, whereas under 400 nm (3.10 eV) and 800 nm (1.55 eV) excitation, the cooperative excitation effect was the only way to form a nucleus, thereby limiting the density of *D*_A_ structures to a lower level.

Nishioka et al.^22^ calculated the threshold excitation energy imparted to an e–h pair to form a new interlayer bond at several lattice sites around the nucleus (process 2). The authors demonstrated a significant reduction in the threshold energy with decreasing distance between the pre-existing interlayer bond and the e–h localised site. ${E}_{\mathrm{e}}$ as low as 1.8 eV was sufficient to form a second interlayer bond at lattice sites adjacent to the pre-existing interlayer bond. Owing to the reduced threshold energy, interlayer bonding tends to occur at atomic sites adjacent to a pre-existing nucleus, resulting in an efficient proliferation of the interlayer-bonded domain from the nucleus. Under 400 and 800 nm excitation, process 2 can determine the geometrical features of the nanostructures, whereas process 1 does not work effectively to form interlayer bonds. In addition, it is strongly suggested that process 2 was associated with a reduced formation rate of *D*_A_-type structures and the rapid aggregation of interlayer bonds for 400 and 800 nm excitation observed at higher total site densities (Fig. 3d,e).

**Figure 4:**
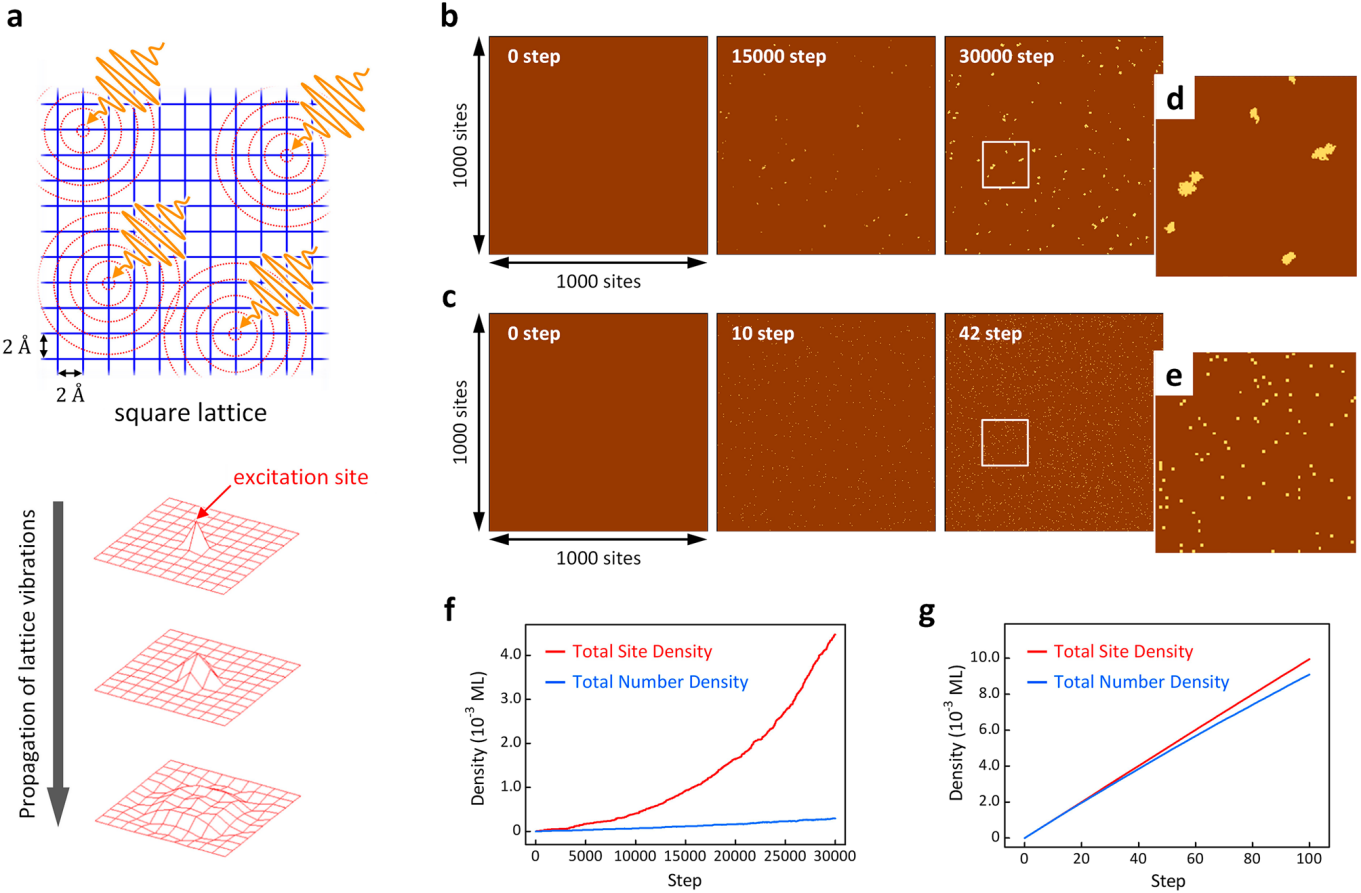
Method and results of the kinetic Monte Carlo simulation. (**a**) Schematic illustration of the calculation model representing random excitations and the following propagations of lattice vibrations on a graphite surface simplified as a square lattice. (**b**,**c**) Snapshots representing morphological evolutions of interlayer bonded domains simulated with 1.55 eV (**b**), and 4.66 eV (**c**), excitations. (**d**,**e**) Expanded images of areas indicated by white squares in (**b**) and (**c**). (**f**,**g**) Total site density (red) and total number density (blue) of the interlayer bond as a function of calculation step excited with 1.55 eV (**f**), and 4.66 eV (**g**).

The previously discussed dynamic pathways for interlayer bond formation at intrinsic lattice sites and sites adjacent to pre-existing interlayer-bonded sites account for the photon energy-dependent growth mode of the *diaphite* domain, as shown in Fig. 3d,e. To verify the proposed mechanism, the morphological evolution of the photoinduced structures under repeated irradiation was simulated using the kinetic Monte Carlo (KMC) method. In general, MD calculations are used for examining the dynamic process of interlayer bond formation^21,22^. However, to simulate the induced morphological evolution, examining various competing reactions among the pre-existing interlayer bonds and excitation sites over a wide surface area and the long-time domain is necessary. Therefore, the MD calculations were performed only for the model parameterisation of the KMC simulation. Figure 4a shows a schematic illustration of the model used in the present KMC simulation, in which the graphite structure is simplified into two layers of a square lattice. The lattice sites where the e–h pairs were localised in the simulation were randomly determined. The propagation of the lattice vibrations induced around the excitation sites and the subsequent interlayer bond formation (or rupture of the pre-existing interlayer bonds) were simulated based on the parameters derived from the MD calculations (see method and supplementary information for the details).

Figure 4b,c show snapshots of the surface morphological evolution simulated with 1.55 and 4.66 eV excitations, respectively. For the two-photon energies, the number of interlayer bonds (bright dots) increased with increasing excitation events. Magnified images (Fig. 4d,e) of the areas indicated by white squares in Fig. 4b,c show aggregated interlayer bonds at 1.55 eV (800 nm) excitation, while they were formed in isolation at 4.66 eV (266 nm) excitation. Figure 4f,g show the total site density of the interlayer bonds and the total number density of the interlayer-bonded domains formed in the model space, respectively, plotted as a function of the number of excitation events. In Fig. 4f, for 1.55 eV excitations, the total site density (red curve) increases acceleratively with increasing excitation events and becomes approximately 15 times higher than the total number density (blue curves). These results indicate that interlayer bonds were efficiently formed at the proximity sites of pre-existing interlayer bonds, and thus the growth of aggregations preferentially occurred. For the 4.66 eV excitation (Fig. 4g), the total site density increased along with the total number density of domains at an early excitation stage and showed a slight deviation at higher doses. This tendency implies that most of the interlayer bonds are isolated at intrinsic lattice sites; that is, the formation of nuclei is the dominant process under 4.66 eV excitation.

**Figure 5:**
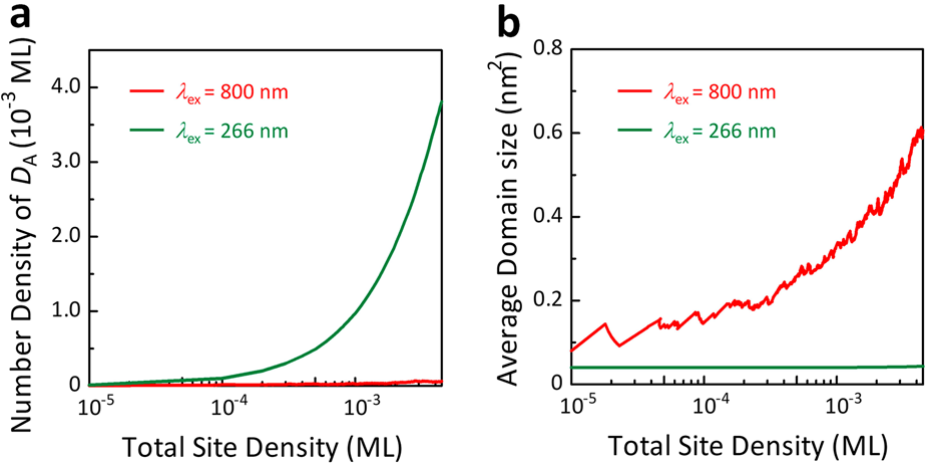
Growth of interlayer-bonded domain in graphite calculated by kinetic Monte Carlo simulation. (**a**) Number densities of *D*_A_ as a function of total site density simulated with 1.55 eV (red) and 4.66 eV (green) excitations. (**b**) Average domain sizes of the nanostructures as a function of total site density simulated with 1.55 eV (red) and 4.66 eV (green) excitations.

Figure 5a,b show the calculated number density of *D*_A_ structures and the average size of the interlayer-bonded domains formed in the model space, respectively, plotted as a function of the total site density of the photoinduced nanostructures for 266 and 800 nm excitation. These calculated results qualitatively reproduce the photon energy-dependent morphologies obtained by the STM experiments (Fig. 3b,c). Therefore, the present simulation explains the crucial role of excitation photon energy in dominating morphological evolution in the G-to-D transition. However, the calculated results provide enhanced yields of *D*_A_ and reduced yields of aggregates of interlayer bonds. As mentioned above, excitation energy was randomly imparted to the lattice sites in the simulation. This assumption is reasonable at the early excitation stage, where photoexcited e–h pairs can be predominately self-localised at intrinsic lattice sites because of the limited number of pre-existing interlayer bonds^21^. However, with increasing excitation events, the increased number of photogenerated interlayer bonds can act as effective trap centres for free e–h pairs. At this stage, the assumption of random excitation is no longer appropriate; however, the trapped e–h pairs enhance the formation rate of interlayer bonds at the proximal sites of pre-existing interlayer bonds. Thus, the calculated results underestimated the number of aggregates of interlayer bonds, which could be one of the reasons for the observed discrepancy between the experimental and calculated results.

Another discrepancy between the experimental and calculated results is that the estimated total site density of the interlayer bonds is two orders of magnitude larger than the experimental value. It should be noted that the present calculation model excludes several effects. First, the thermal effect is an essential factor that should be considered. In general, the increase in sample temperature under laser excitation was attributed to the optical absorption of the materials. Under the present excitation conditions, the interlayer bond formation was induced without causing any surface damage, such as laser ablation or delamination of top layer^35^, and the maximum temperature was below the sublimation temperature of graphite in a UHV (1800 K^36^). Nevertheless, some existing interlayer-bonded structures (*D*_A_, *D*_B_, and *D*_C_), stable at room temperature, should have been efficiently relaxed to their ground states by the laser-induced heating. This thermal effect is expected to reduce the net product of the interlayer bonds. Second, for 4.66 eV light, the excitation can promote rupture of the pre-existing interlayer bonds owing to the enormous excitation energy imparted (see supplementary information). Therefore, the competition between the formation and rupture of interlayer bonds occurs in a practical situation. Although the present simulation also considered laser-induced bond rupture, the effective trapping of e–h pairs by pre-existing interlayer bonds further enhanced the bond rupture around the pre-existing interlayer bonds. The abovementioned effects allow for suppressing the growth of interlayer-bonded domains; therefore, a quantitatively better evaluation would be provided by assembling these effects into the simulation.

In summary, we obtained atomic-scale views of several nanoscale structures formed during the photoinduced G-to-D structural phase transition and revealed the fundamental processes involved in forming *sp*^3^-like interlayer-bonded domains. The primary process involved the formation of a nucleus composed of two interlayer bonds with a relatively weak *sp*^3^-bonding nature. The subsequent aggregation of the interlayer bonds around the nucleus resulted in the growth of the *diaphite* phase, where *sp*^3^-like interlayer bonds were organised periodically, together with striking in-plane and out-of-plane lattice displacements. Two different dynamic pathways have been proposed to explain the photon energy-dependent growth modes of the *diaphite* phase. While the present study provides the atomic scale views of typical nanostructures generated in the processes of photoinduced G-to-D structural transition, direct tracking the growth and *sp*^2^–*sp*^3^ conversion process could be further investigated using methods such as STM analysis at cryogenic temperature and Raman spectroscopy. Both of these techniques would offer more direct evidence and remains a promising avenue for future work. Nevertheless, the present findings provide insight into the optical control of *sp*^2^–*sp*^3^ conversion and the organisation of nanoscale structures in graphene-related materials, which not only deepen our understanding of light-matter interactions but also advance innovative material processing techniques.

## Methods

### Experimental setup

The experiments were performed at room temperature in a UHV chamber (base pressure of 7 × 10^−9^ Pa). Flat and clean surfaces of HOPG were obtained by peeling the upper layers with adhesive tape, and the sample thus prepared was mounted on the STM stage (Unisoku, USM-901). Femtosecond laser pulses were generated at a repetition rate of 1 kHz using mode-locked Ti: sapphire and a regenerative amplifying system (Spectra-Physics, Hurricane). The laser pulse duration was 120 fs. Laser light with several photon energies (800 nm fundamental, frequency-doubled (400 nm), and frequency-tripled pulses (266 nm)) was used to excite the HOPG surface. The laser pulses were focused on the sample placed on the STM stage at an incident angle of 45° from the surface normal. By combining a half-wave plate and polariser, the polarisation and intensity of the laser pulses were precisely controlled. Laser-induced structural changes were characterised in situ at room temperature through STM using a mechanically etched Pt–Ir tip. The images were captured both in constant current mode and quasi-constant-height mode. The LDOS near the Fermi level was measured using scanning tunnelling spectroscopy (STS) by sweeping the sample bias voltage from − 1.0 to 1.0 V. The obtained STM and STS data were analysed by using WSxM and Gwyddion software.

### Theoretical calculations

The morphological evolution of graphite under 1.55 and 4.66 eV laser excitations was simulated using combined MD and KMC methods. MD calculations using the Brenner potential^37^ were first performed for the model parameterisation of the KMC simulation. The system consisted of two graphene layers with AB stacking, and the interlayer distance was set to 3.35 Å. Each layer includes 6240 carbons in an area of about 12.8 × 12.8 nm with imposed periodic boundary conditions. The calculation assumed self-localisation of the photoexcited e–h pairs at the excited sites^21,22^. The excess excitation energy, depending on the excitation photon energy, was set to promote local lattice distortion and the subsequent propagation of lattice vibration around the excited site. By systematically setting the excited sites, the time evolution of the lattice vibrations was calculated to be up to 1 ps. The calculation results were analysed to extract the model parameters (see supplementary information) for the subsequent KMC simulation. KMC was performed to simulate the morphology of the graphite surface under repeated laser-pulse irradiation. The graphite structure was reduced to two graphene layers, wherein each hexagonal lattice was mapped to a 1000 × 1000 square lattice (200 × 200 nm area including 10^6^ carbon atoms) with boundary conditions. At each calculation step, 100 excitation sites (excitation density of 0.0001) were roughly estimated based on the experimental laser flux and the self-localisation probability of the e–h pair theoretically predicted in Ref.^21^ and chosen randomly. The spherical waves of lattice vibration around the excited sites were generated. The lattice vibration parameters were obtained from the results of the MD calculations (see supplementary information). The spherical waves were superimposed to calculate the out-of-plane lattice displacements at all sites in the model space. The interlayer bond formation and the rupture of pre-existing interlayer bonds were determined based on the interlayer distance and the related energy potential proposed by Brenner^37^. After the pre-set KMC steps, the surface morphologies were statistically analysed.

### Supplementary Information

Supplementary Information.

Supplementary Video S1.

Supplementary Video S2.

Supplementary Video S3.

Supplementary Video S4.

Supplementary Video S5.

Supplementary Video S6.

Supplementary Video S7.

Supplementary Video S8.

Supplementary Video S9.

Supplementary Video S10.

## Data Availability

The datasets used and/or analysed during the current study available from the corresponding author on reasonable request.
