# Supplementary material for: Atomic-scale view of the photoinduced structural transition to form sp3-like bonded order phase in graphite
Source: Sci Rep. 2023 Dec 15;13:21439. doi: 10.1038/s41598-023-47389-x (PMC10724284; doi:10.1038/s41598-023-47389-x)
Supplement: Supplementary file 1 — Supplementary Information. [file 41598_2023_47389_MOESM1_ESM.pdf]

Supplementary Information

Atomic-scale view of the photoinduced structural transition to form sp^3 -like bonded order phase in graphite

Eiichi Inami[1*] , Keita Nishioka[2], and Jun'ichi Kanasaki[3]

[*]e-mail: inami.eiichi@kochi-tech.ac.jp

[1]School of Systems Engineering, Kochi University of Technology, 185 Miyanokuchi, Tosayamada, Kami, Kochi 782-8502, Japan

[2]Math. and Science Education Research Center, Kanazawa Institute of Technology, 7-1 Ohgigaoka, Nonoichi, Ishikawa 921-8501, Japan

[3]Graduate School of Engineering, Osaka Metropolitan University, 3-3-138 Sugimoto, Sumiyoshi-ku, Osaka 558-8585, Japan

Contents

- Supplementary Notes

- Supplementary Figures

- Supplementary References

- Supplementary Videos

Supplementary Notes

SN1. Molecular dynamics simulation (single-site excitation):

Morphological evolution in the photoinduced graphite-to-*diaphite* (G-to-D) transition was simulated using kinetic Monte Carlo (KMC) method with parameters determined through molecular dynamics (MD) simulation[1,2]. In this Note, we present the results of MD simulation focused on the simplest scenario of single-site excitation, where a specific amount of energy was imparted to a pair of α-carbons sitting face-to-face in the adjacent graphene layers of pristine graphite.

In the MD simulation, we assumed a bilayer graphite model system in which the two layers are connected with a single interlayer bond between a pair of nearest α-carbons. The potential energy was calculated as a function of the interlayer bond length using the semiempirical Brenner's potential (refer 3 and Methods section in the main text for a detailed procedure). Fig. S2 shows the potential energy as a function of interlayer bond length, referenced to the state of pristine graphite. The result exhibits a local minimum energy at the bond length of approximately 1.56 Å, indicating the metastable state involving a single interlayer bond configuration. Additionally, Fig. S2 reveals an energy barrier of approximately 0.8 eV to be overcome in the formation process of the single interlayer bond. However, our previous MD simulation using Brenner's potential[1] reported that an excitation energy higher than 4.5 eV is necessary for single-site excitation to form an interlayer bond, because most of the excitation energy imparted to the carbon pair is dissipated into the surrounding carbons via lattice vibrations (see Video S8).

We examined the energetic condition mentioned above for two excitation photon energies used in our experiments (1.55 eV and 4.66 eV). Videos S1 and S2 show the calculated lattice vibrational dynamics after single-site excitation at 1.55 eV and 4.66 eV, respectively. It is evident that the 1.55-eV excitation never lead to the formation of any interlayer bonds, whereas 4.66-eV excitation induces the formation of an interlayer bond at the excitation site. We also analyzed in Fig. S3 the time evolutions of the displacements of the two excited carbons along the *c*-axis of graphite (along out-of-plane direction) for both excitation energies. As shown in Fig. S3, the two paired carbons in both cases initially approach each other, followed by the damped oscillatory motion. The interatomic distance eventually converged to 3 Å for 1.55 eV, and to 1.56 Å for 4.66 eV, around 1.0 ps after the excitation. Consequently, excitation with 4.66 eV causes the formation of a single interlayer bond with a length of 1.56 Å, close to the bond length in diamond, 1.54 Å. On the other hand, excitation with 1.55 eV does not result in interlayer bond formation, eventually returning to the pristine graphite.

SN2. Molecular Dynamics simulation (cooperative effect):

In the present KMC method, excitation by single laser pulse was modeled as follows: a predetermined number of α-carbon pairs are randomly selected in the model space and excited simultaneously. This procedure was repeated to model the repeated excitation with laser pulses. Indeed, such "repeated multisite excitations" cause several cooperative phenomena that facilitate interlayer bond formation with lower photon energy compared to the case of single-site excitation (4.5 eV, see SN1)[2]. In this Note, we demonstrate two typical cooperative phenomena using MD simulation.

Video S3 shows an example of cooperative phenomena where three proximal α-carbon pairs are simultaneously excited with an energy of 2.51 eV/pair. As noted in SN1, the excitation energy (2.51 eV/pair) is insufficient to form an interlayer bond under single-site excitation condition. However, in this case, lattice vibrations induced by the excitations of α-carbon pairs propagate from the excited sites and interfere with each other around the excited sites, resulting in the enhanced oscillation amplitudes at the central site. This cooperative phenomenon leads to the formation of an interlayer bond at the site.

Video S4 shows another example of cooperative phenomena where an energy of 2.0 eV is given to an α-carbon pair near a pre-existing interlayer bond leading to the formation of a new single interlayer bond at the excited site. Although the imparted energy is insufficient to form an interlayer bond at perfect graphite sites, the paired carbon atoms adjacent to pre-existing interlayer bond effectively get closer to form an additional interlayer bond. It is noteworthy that the processes depicted in Video S3 and Video S4 correspond to the "nucleation" and "proliferation" of interlayer bonded phase, respectively.

SN3. Molecular dynamics simulation (cooperative effect under 1.55-eV excitation):

As described in SN1 and SN2, threshold energy for the interlayer bond formation is more than 4.5 eV in the single-site excitation case, while the cooperative effect drastically reduces the threshold energy down to 2.51 eV for nucleation and 2.0 eV for proliferation. However, the experimental results in the main text demonstrate the interlayer bond formation is possible even at the lower excitation energy of 1.55 eV. To confirm the cooperative effect associated with the bond formation by 1.55 eV excitation, we performed MD simulation using the following procedure.

In each calculation step for a single pulse excitation, twelve α-carbon pairs were randomly selected within the model space (128 Å × 128 Å) to impart an energy of 1.55 eV/pair. The subsequent lattice vibrational dynamics were then calculated for a duration of 1 ps. After 1 ps, we applied external damping forces proportional to the oscillating carbon velocities for an additional 1 ps to attenuate the lattice vibration. These calculation procedures were repeated for the predetermined number of cycles to simulate the repeated laser pulse excitations. Fig. S4 (and Video S5) presents the snapshots of the morphological evolution obtained through the aforementioned procedure. The number of interlayer bonds increases with increasing calculation steps, and the result clearly indicates that an excitation energy of 1.55 eV is still sufficient to induce the cooperative effect.

The events of interlayer bond formation by 1.55 eV excitation were very limited. To confirm the cooperative effects, we used the excitation density of 0.002 in the present calculation, which is significantly higher than the experimental conditions It is noted, therefore, that the calculated morphologies in Fig. S4 (and Video S5) should be different from the experimentally obtained ones. The morphological changes under the appropriate excitation density were calculated using KMC, and the results will be described in SN8.

SN4. Molecular dynamics simulation (excessive excitation effects):

As shown in Video S2, the single-site excitation with energy of 4.66 eV can induce a new interlayer bond at the excitation site, leading to the formation of a nuclear in graphite. However, the excitation energy of 4.66 eV is excessively high, thereby the excitation also breaks the pre-existing interlayer bond in certain cases. Furthermore, in other cases, such excessively high excitation energy prevents the interlayer bond formation by cooperative effects (see SN2). In this Note, we employ MD simulation to demonstrate typical examples of these "excessive excitation effects." Video S6 presents an example where an excitation energy of 4.66 eV is imparted to an α-carbon pair forming an interlayer bond. In this case, the excitation first brought the two carbons closer to each other, then, the atoms repelled their counterparts with substantial kinetic energy, resulting in the rupture of the interlayer bond. This excitation-induced bond rupture corresponds to a kind of laser-induced relaxation from *diaphite* to graphite. Video S7 shows another example where the excitation energy of 4.66 eV was imparted to an α-carbon pair adjacent to a pre-existing interlayer bond. Similarly to the case in Video S6, the excitation induced the interlayer compression followed by the repulsion between the two carbons. The process caused by the excessive excitation is a probable explanation why the proliferation is less promoted under 4.66-eV excitation, compared to the case by 1.55-eV excitation (refer to the main text).

SN5. Molecular dynamics simulation (lattice vibrational dynamics in pristine graphite):

As explained in SN1-4, imparted energy causes lattice vibration around the α-carbon pair, and interlayer bond is formed when the carbon atoms get close each other to a specific distance. Therefore, the laser-induced vibrational dynamics is a key process to be adequately treated in the present KMC simulation of the photoinduced G-to-D transition. Here we detail the procedure of MD simulation used to extract crucial parameters of lattice vibration associated with interlayer bond formation and/or rupture. In the MD simulation, we calculated the vibrational dynamics of bilayer graphite after single-site excitation with energies of 1.55 eV and 4.66 eV. Following excitation at the central site of the model space, the induced lattice vibration propagates concentrically as a spherical wave, of which the amplitude depends on the excitation energy imparted. In the case of multisite excitation (refer to SN2), the excitation sites were randomly selected, and the induced lattice vibrations propagate from these selected sites. Therefore, the amplitude of vibration waves superimposed at each site depends not only on the excitation energy but also on the spatial relationships between the excitation sites. Video S8 shows the time evolution of spherical waves within a lower atomic layer of a bilayer graphite (20 Å × 20 Å region) after excitation at the central sites. In the present KMC simulations, the amplitude of the spherical waves in graphite lattice (middle) were projected onto a square lattice (right), as described in SN8.)

SN6. Molecular dynamics simulation (vibrational dynamics in graphite including interlayer bonds):

In SN2, we described the effects of repeated multisite excitations of the bilayer graphite, which includes interlayer bonds connecting the two layers. Due to different local configurations of carbon pairs, the dynamics of laser-induced vibration should be different at perfect lattice sites, interlayer bonded sites, and sites near the interlayer bond. Thus, it is essential to take into account the site-dependent lattice dynamics to determine several parameters used in the KMC method. In this Note, our focus is paid on the MD simulation to calculate vibrational amplitudes at the perfect lattice site and the interlayer bonded site.

The elastic constant associated with the excitation-induced vibrations is governed by the bonding configuration of α-carbon pair. Here, an α-carbon pair at a perfect lattice site, that is non-interlayer-bonded pair, is labeled as C_1 and an interlayer-bonded α-carbon pair is labeled as C_2. We define $z_1(t)$ and $z_2(t)$ as the time-dependent out-of-plane displacements of each carbon atom within C_1 and C_2 when the same excitation energy E_{ex} is imparted. $z_1(t)$ and $z_2(t)$ can be expressed under the simple harmonic approximation as

$$z_i(t) = Z_i \cos\left(\frac{2\pi}{T_i} t + \theta_i\right) \qquad (i = 1, 2), \tag{1}$$

with

$$Z_i = \sqrt{\frac{2E_{\mathrm{ex}}}{k_i}} \qquad (i = 1, 2), \tag{2}$$

where Z_i, T_i, θ_i and k_i represent vibrational amplitude, vibrational period, initial phase and elastic constant of C_i, respectively. Then, we obtain the ratio of the vibrational amplitudes from Eqs. (1) and (2) as:

$$\frac{Z_2}{Z_1} = \sqrt{\frac{k_1}{k_2}} = \frac{T_2}{T_1}\ . \tag{3}$$

Therefore, the ratio of the vibrational amplitude (Z_2/Z_1) under excitation with certain energy can be obtained by evaluating the ratio of the oscillation period. Fig. S5 displays the results of MD simulation, which calculated time evolution of displacements along the *c*-axis of the lower carbon in the pair when an excitation energy of 0.1 eV is imparted to the perfect lattice site (left) and the interlayer-bonded site (right). In both cases, carbons initially come closer to each other, followed by damping oscillations. However, their oscillation periods were clearly different: 0.125 ps for the perfect lattice site and 0.0353 ps for the interlayer bonded site. From these results, Z_2/Z_1 was estimated to be approximately 0.282.

SN7. Molecular dynamics simulation (critical carbon distances for interlayer bond formation):

As is explained in SN1-4, interlayer bond formations occur when excitation-induced lattice vibrations promote the α-carbons in pair to approach each other within specific distance range (green shaded region in Fig. S2). In this Note, we detail the MD simulation to analyze the critical distance for the interlayer bond formations.

Left-hand panel of Fig. S6 represents the time evolution of the displacements for two α-carbons sitting face-to-face in the neighboring atomic layers of pristine graphite when energies of 4.5 eV (red dashed curve) and 4.6 eV (green solid curve) are imparted to the paired atoms. For excitation of 4.5eV, the two carbons initially approach each other but then gradually move apart accompanied with oscillations. On the other hand, the 4.6 eV excitation decreases the distance between the two carbons to form an interlayer bond. It should be noted that the critical distance is 1.94 Å, where the curves for the 4.5 eV and 4.6 eV excitations deviate from each other. Thereby, this suggests that the bond length of 1.94 Å is the upper threshold d_U for interlayer bond formation.

Similarly, right-hand panel of Fig. S6 represents the results for the single-site excitations with energies of 5.1 eV and 5.2 eV. The 5.1-eV excitation results in the interlayer bond formation, while the 5.2 eV excitation does not. The displacement curves for 5.1-eV and 5.2-eV excitations deviate at the bond length of 1.26 Å. Thus, we regarded the bond length of 1.26 Å as the lower threshold d_L below which the contraction of the α-carbon pair prevents interlayer bonding.

SN8. Procedure of the kinetic Monte Carlo simulation:

Based on the parameters determined by the MD simulation (refer to SN5-7), the morphological evolution of the photoinduced G-to-D transition was simulated using the KMC method. This Note details the procedure in the present KMC method. The scheme of the KMC simulation is shown in Figs. S7. In our model space, the graphite structure was simplified to two carbon layers. Planar hexagonal lattice was replaced onto a 1000×1000 square lattice (corresponding to a 200 nm $\times$ 200 nm area containing 10^6 lattice sites) under periodic boundary conditions. It is important to note that the utilization of such a simplified but extensive model space is one of the key advantages of employing the KMC simulation. This enables the simulations at lower excitation density comparable to the experimental conditions, along with the reduced computational cost.

Morphological change induced by a single laser pulse was simulated in each calculation step according to the following procedure. Firstly, within the model space, 100 excitation sites (corresponding to an excitation density of 0.0001) were randomly selected. The excitation density was estimated based on the experimental laser flux and the theoretically predicted self-localization probability of a photogenerated electron-hole pair[1]. At each excitation site, a spherical wave representing the excitation-induced lattice vibration was generated, and its propagation was calculated by using the results of the MD simulation (refer to Video S8). By superimposing the individual spherical waves generated at excitation sites, the out-of-plane displacement of the carbon was calculated at each site in the model space. In this procedure, we incorporated the displacements dependent on the excitation energy and site-dependent elasticity determined based on the results of the MD simulation (refer to SN5 and SN6). The displacement at interlayer-bonded sites was determined by multiplying the displacement induced at perfect lattice sites by the ratio as described by Eq.(3) in SN6 ($Z_2/Z_1 \approx 0.282$). The resultant configuration, formation of a new interlayer bond or the rupture of a pre-existing interlayer bond, was determined based on the critical bond lengths between two carbons calculated by the MD simulation: an interlayer bond is formed if the bond length falls within the range between d_{L} and d_{U}, as described in SN7. This calculation step was repeated for a predetermined number of cycles to simulate the morphological evolution under repeated laser pulse excitations.

SN9. Results of the kinetic Monte Carlo simulation:

Following the procedure described in SN8, we conducted KMC simulations with excitation energies of 1.55 eV and 4.66 eV. Video 9 (for 1.55eV) and Video 10 (for 4.66 eV) show the movies, for which the calculation steps set to 30000 for 1.55 eV and 50 for 4.66 eV, respectively. To analyze the morphologies, we employed the same statistical method as described in the main text. In this method, the configuration composing of more than two interlayer bonds formed at adjacent lattice sites was regarded as a single domain. Note that isolated interlayer bonds were counted as individual domains with the size of 1. The results of the statistical analysis are referenced in the main text.

Supplementary Figures

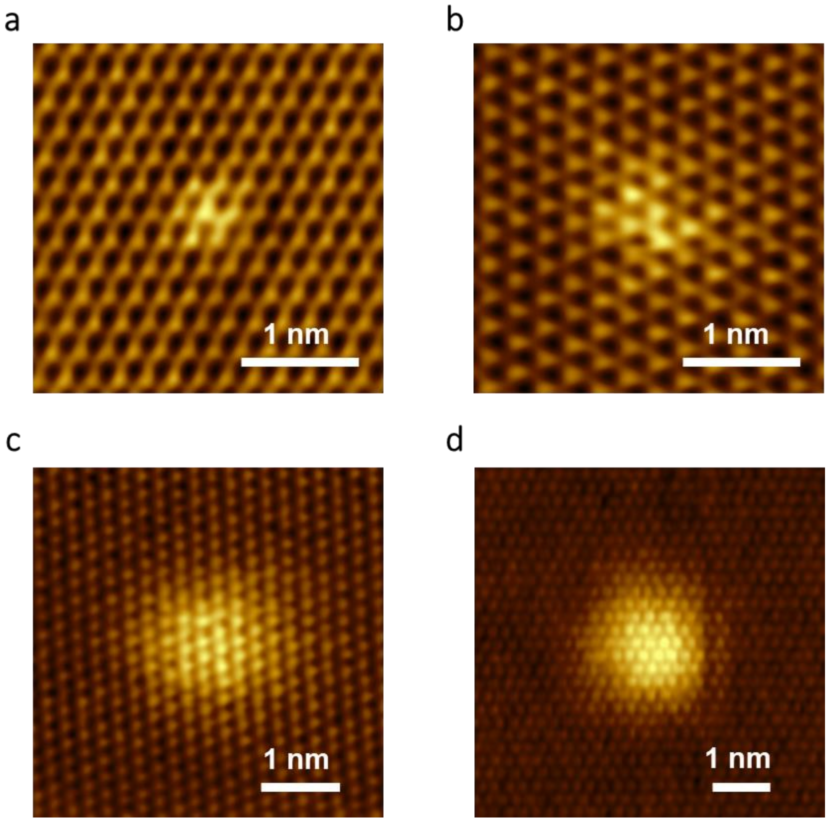

Fig. S1: STM topographic images of photoinduced domains on graphite surface. All domains were classified as D_B-type structure (see the main text for details). Sample bias was set at V_S= 0.1 V for image **a**, V_S= − 0.1 V for image **b**, V_S=0.3 V for image **c**, V_S= 0.2 V for image **d**.

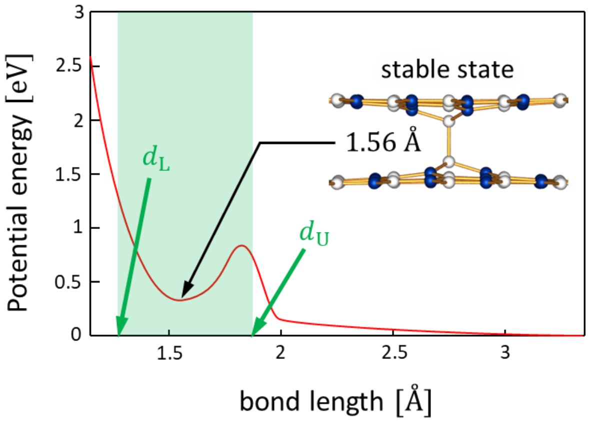

Fig. S2: Potential energy profile between pristine graphite and graphite including a single interlayer bond by an α-carbon pair, calculated by Brenner's semiempirical potential. The energy is referenced to the pristine graphite state and plotted as a function of interlayer bond length between the two paired carbons. Green shaded area between d_L and d_U is the distance range where interlayer bond is formed (refer to SN7).

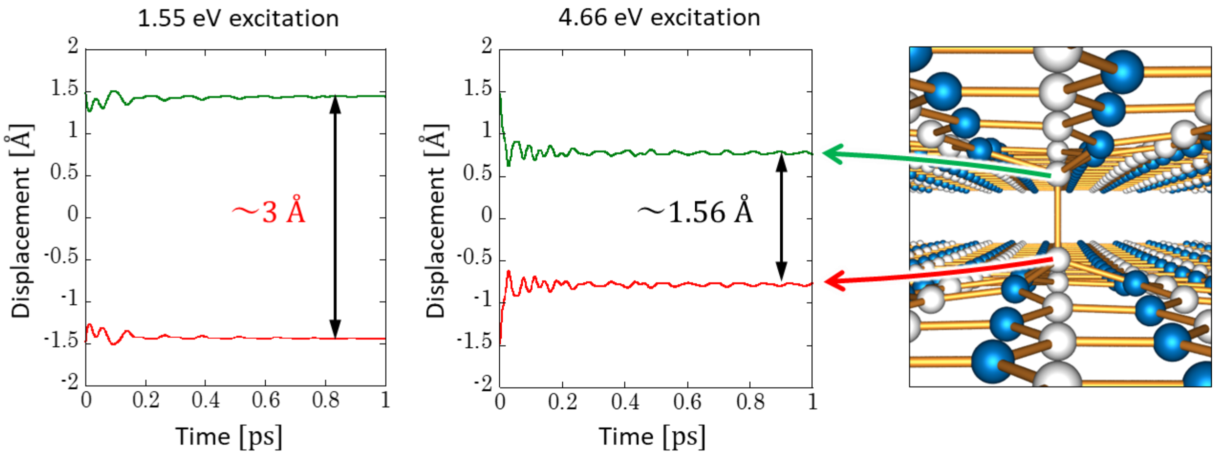

Fig. S3: Time evolution of the displacements of two paired α-carbons along the *c*-axis (perpendicular to the graphite surface) following 1.55-eV (left) and 4.66-eV (center) excitation. The displacements are referenced to the middle point between the two graphene layers. The right-hand figure depicts a snapshot of the MD calculation taken at 1.0 ps after the 4.66 eV excitation.

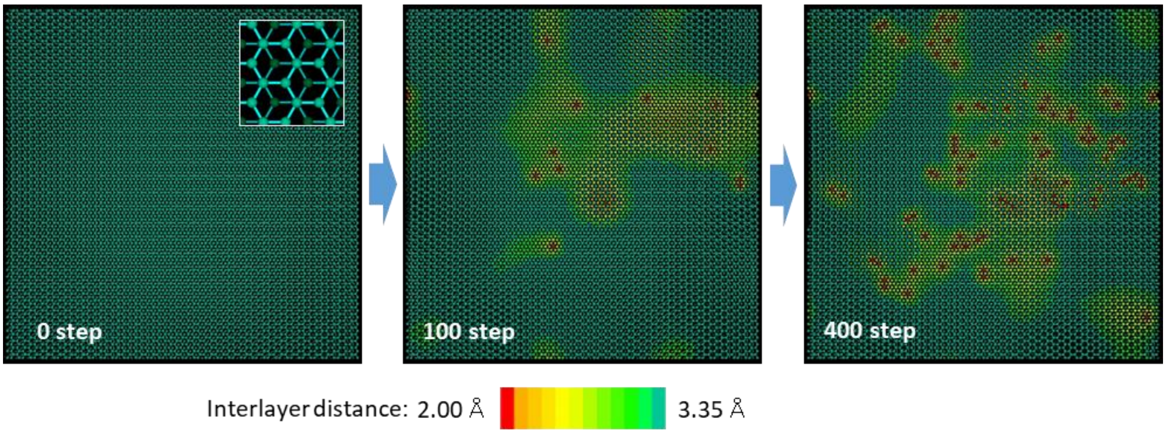

Fig. S4: Snapshots of the excitation-induced morphological evolution on graphite, obtained through MD simulation. The initial state (0 step) consists of a bilayer graphite lattice with the size of 128 Å × 128 Å (containing 12480 carbons). The images labeled as 100 and 400 steps depict the surfaces after 100 and 400 cycles of random multisite-excitation (see SN2 for details). The inset in the left-hand figure provides an enlarged view of the bilayer graphite lattice. Carbon atoms are colored according to their interlayer distances using the palette at the bottom. Red color point indicates the site where an interlayer bond is formed.

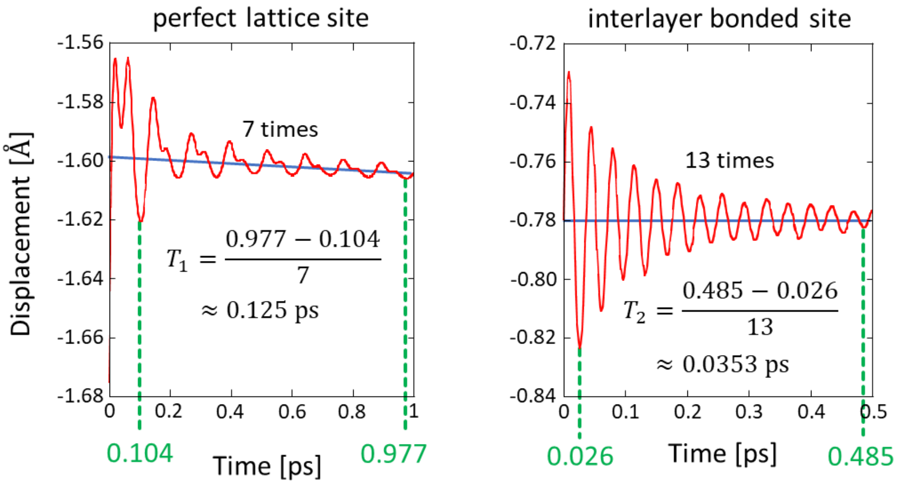

Fig. S5: Time evolution of displacements of an α-carbon at perfect lattice site (left) and an interlayer bonded site (right) in the lower layer of bilayer graphite, after excitation energy of 0.1 eV are imparted to the sites.

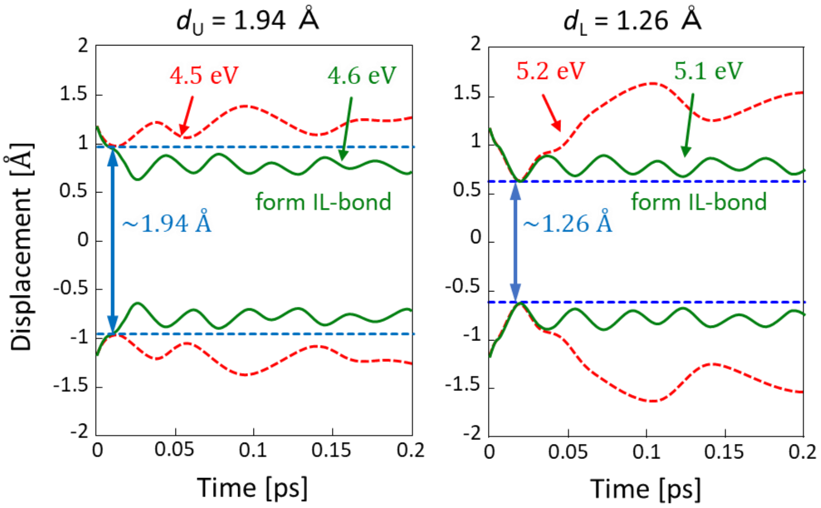

Fig. S6: Time evolution of displacements of two paired carbons, depicted in Fig. S3. The left-hand figure corresponds to the case for excitation energies of 4.5 eV and 4.6 eV, while right-hand one corresponds to the case for excitation energies of 5.1 eV and 5.2 eV. The bond lengths labeled as d_U=1.94 Å (left) and d_L= 1.26 Å (right) represent the upper and the lower critical distances for interlayer bond formation (see SN7).

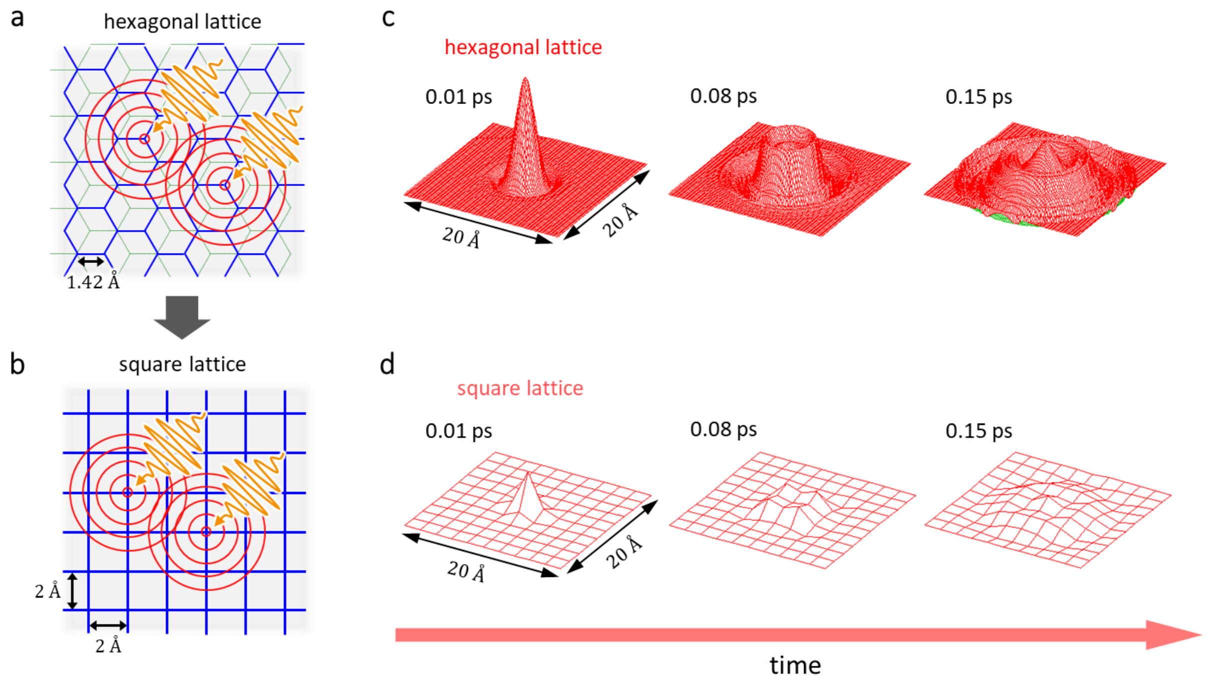

Fig. S7: Mapping a bilayer graphite from hexagonal lattice, a and c, to a simplified square lattice, b and d. The lattice constant of the square lattice is set to be 2 Å. a, c, Superposition of spherical waves generated at lattice sites. c, d, Snapshots of propagation of a spherical wave generated at a single lattice site after excitation with an energy of 4.66 eV.

Supplementary Videos

Video S1: Result of the MD simulation, demonstrating lattice vibrational dynamics of a bilayer graphite after single-site excitation where an excitation energy of 1.55 eV is imparted to an α-carbon pair (red colored atoms).

Video S2: Result of the MD simulation, demonstrating lattice vibrational dynamics of a bilayer graphite after single-site excitation where an excitation energy of 4.66 eV is imparted to an α-carbon pair (red colored atoms).

Video S3: Result of the MD simulation, demonstrating lattice vibrational dynamics of a bilayer graphite after multisite excitation where an excitation energies of 2.51 eV/pair are imparted to three α-carbon pairs (red colored atoms).

Video S4: Result of the MD simulation, demonstrating lattice vibrational dynamics of a bilayer graphite after excitation energy of 2.0 eV is imparted to an α-carbon pair (red colored atoms) adjacent to the interlayer bonded site.

Video S5: Result of the MD simulation demonstrating morphological evolution of a bilayer graphite after repeated random multisite excitation with an energy of 1.55 eV. The video continuously displays the morphology up to 400 steps, with each step showing a progression of 5 steps. Carbon atoms are colored by their interlayer distances, with red indicating the presence of interlayer bonds.

Video S6: Result of the MD simulation, demonstrating the effect of excitation on a α-carbon pair (red colored atoms) forming interlayer bond in bilayer graphite. The excitation energy is set to be 4.66 eV.

Video S7: Result of the MD simulation, demonstrating the effect of excitation on a α-carbon pair (red colored atoms) adjacent to the interlayer bonded site in bilayer graphite. The excitation energy is set to be 4.66 eV.

Video S8: Result of the MD simulation demonstrating time evolution of a lattice vibration after single-site excitation with energies of 1.55 eV (top) and 4.66 eV (bottom) at a surface site. The left side graphs profile the displacements perpendicular to the surfaces are profiled as a function of the distance from the excited site. The center and right graphs depict the lattice vibrational dynamics mapped on a bilayer graphite surface (center) and a square lattice (right) with a lattice constant of 2 Å.

Video S9: Result of the KMC simulation demonstrating morphological evolution of interlayer bonded domains after repeated random multisite excitation with an energy 1.55 eV. The video continuously displays the morphology up to 30000 steps, with each step showing a progression of 100 steps. The pristine graphite sites are colored in brown, while the interlayer bonded sites are colored in bright yellow.

Video S10: Result of the KMC simulation demonstrating morphological evolution of interlayer bonded domains after repeated random multisite excitation with an energy 4.66 eV. The video continuously displays the morphology up to 50 steps, with each step showing a progression of 1 step. The pristine graphite sites are colored in brown, while the interlayer bonded sites are colored in bright yellow.

Supplementary References

1. Nishioka, K. & Nasu, K. Early-stage real-time dynamics of interlayer *sp*^{3}-bond formation by visible-light irradiation of graphite. *Phys. Rev. B* 80, 235420 (2009).
2. Nishioka, K. & Nasu, K. Cooperative domain-type interlayer *sp*^{3}-bond formation in graphite. *Phys. Rev. B* 82, 035440 (2010).
3. Brenner, D. W. Empirical potential for hydrocarbons for use in simulating the chemical vapor deposition of diamond films. *Phys. Rev. B* 42, 9458–9471 (1990).
